# Melatonin and cancer

**Published:** 2014-09-25

**Authors:** AA Zamfir Chiru, CR Popescu, DC Gheorghe

**Affiliations:** * "Grigore Alexandrescu" Hospital, Bucharest, Romania; ** "Colțea" Hospital, Bucharest, Romania; *** "MS Curie" Hospital, Bucharest, Romania

**Keywords:** melantonin, cancer, redox processes, inhibitor, receptors

## Abstract

Abstract

Melatonin plays an important role in cancer (tumor growth and metastasis) through different pathways and may have therapeutic significance.

 Melatonin, known as N acetyl-5-methoxytryptamine is a hormone produced by the pineal gland and other organs as well: skin, retina, bone marrow [**1**]. Melatonin’s serum levels have showed the involvement of this hormone in circadian rhythm [**2**]. Its secretion is stimulated by darkness and decreased by light, serum levels peaking between 2 a.m. and 5 a.m.[**2**].

 Melatonin produces its biological effects through melatonin receptors [**3**]. 3 types of receptors, expressed in various amounts by different tissues of the body [**4**] have been described. Sometimes, the effects can be mediated through interaction with different cell kinases [**5**]. Melatonin is involved in redox processes of the cells, acts directly at the augmentation of Natural Killer cell activity, stimulates cytokine production (IL-2 and IL-6) [**6**] and protects hematopoietic precursors from the toxic effect of chemotherapy and radiotherapy [**6**].

 Several epidemiologic studies revealed that the disruption of normal circadian rhythm may increase the risk of developing cancer (especially those hormone-dependent) [**7,8**].

 Studies on the effect of melatonin on tumor growth and angiogenesis in xenograft model of breast cancer revealed that melatonin reduces tumor growth and cell proliferation [**9**] and inhibits angiogenesis [**10**] by decreasing the expression of VEGF receptor 2 [**11**] and increasing the expression of epidermal growth factor receptor and insulin growth factor 1. It has also been shown that melatonin participates in the activation of lymphocytes and monocytes/macrophages, preventing tumor development [**12**].

 Also, the pineal gland hormone inhibits tumor invasion by blocking stromal derived factor 1, through p-38 pathway (melatonin represses phosphorylation of p-38).

 Melatonin also affects the redox status of the cells [**13**] and that is an important part in regulating activities of the matrix metalloproteinases (MMPs). Gelatin zymography or Western immunoblot analysis revealed that the inhibitor effect of melatonin is centered on the activity and expression of MMP2 and MMP9. This effect is mediated through elevated expression of MT1 receptors, in a dose and time dependent manner. 

 N acetyl 5-metoxytriptamine was also studied on rat model with gastric ulcer. Findings proved an important role, dose-dependent, in reducing activities of MMP2 and TNFα and in increasing expression of tissue inhibitors of metalloproteinases (TIMP1 and TIMP2). The involvement of matrix metalloproteinases and MMP inhibitors, as well as TNF in cancer development is well-known.

 Melatonin reduces the production of gelatinase (that is MMP9) and subsequently its activity through up regulation of MMP 9 specific inhibitor tissue TIMP1, via nuclear factor_kB (NF-kB) translocation and transcriptional activity [**14**]. Also, the same effect on MMP activity is proved by melatonin interaction with Zinc ion, protein 421, alanine 191 and the three histidines that compose the catalytic site residues. In conclusion, melatonin inhibits MMP9’s activity by binding to its active site.

 Melatonin plays an important role in preventing the age-related inflammatory response [**15**] by decreasing the levels of nuclear transcription for lipocalin 2 (Lcn2), a member of the acute phase response family of proteins. The same substance seems to have increased serum levels in various human cancers [**16**], when mixed with matrix metalloproteinase 9. 

 Studies on pinealectomised rats analyzed the expression of inflammatory cytokines and phosphorylation levels of signal pathways of melatonin. Increased expression of matrix metalloproteinase 9, monocyte chemotactic protein-1 (MCP-1), vascular adhesion molecule (VCAM-1) found in pinealectomised rats, were correlated with melatonin deficiency. VCAM-1 molecule seems to be associated with tumor evasion from the immune response [**17**]. Other findings in studies revealed decreased melatonin serum levels of tumor necrosis factor, interleukin-6 and C reactive protein as well as serum triglyceride, very low lipoprotein cholesterol and glucose.

 The protective effect of melatonin on human umbilical vein cells was proved by NF-kB translocation into nucleus. NF-kB activation as well as the up regulation of MMP9 expression and down regulation of TIMP1 induced by IL1β is significantly inhibited by Melatonin [**14**].

 Cell migration and invasion of glioma were associated with basal intracellular free radical generation. Inhibition of MMP2 and MMP9 expression as well as cell migration and invasion effects were explained by using the melatonin’s inhibition of oxidative stress pathway in glioma cells.

 Other findings like the inhibiting of kB nuclear factor pathway activation by pyrrolidine dithiocarbamate and also the suppression of IL-1β-induced adhesion (through induced expression of MMP2, MMP9, CD 45), were demonstrated by other studies.

 In conclusion, melatonin has multiple effects and plays an important role in adhesion, migration, invasion and apoptosis of cells [**13**].

 Some studies about inflammation and tumor genesis revealed that cyclooxygenase-2 (COX2) is overexpressed in cancer cells. Melatonin possesses COX2 suppressing activity [**18**] and inhibits tumor growth and cancer metastasis at supra-pharmacological concentration in vitro, on xenograft model, same as 5-MTP (5-Methoxytryptophan or cytoguardin). These findings are important for future development of cancer chemoprevention.

 The biomodulation of cancer chemotherapy and radiotherapy by using melatonin administration confirms decreased toxicity and increased efficacy of cancer treatment on patients with poor clinical status and metastatic solid tumors [**19**].

 Well-controlled trials, that examined the correlation between melatonin levels and neoplastic activity, concluded that melatonin, through its antiproliferative, antioxidative and immunostimulatory actions, should be considered a naturally oncostatic agent [**20,21**].
